# Dyspepsia and Throat-Ail

**Published:** 1858-06

**Authors:** 


### DYSPEPSIA AND THROAT-AIL.

"Dear Sir:—I do like the candid way in which you treat everything. I go to a physician here, and state my complaint, and he says he *thinks* he can help me, and forthwith prepares 'something to take,' and perhaps tells me what is the matter with me. I get well, but am still ignorant of the cause which made me sick, and, of course, do not know how to avoid the recurrence of the disease again, unless I have wit enough to discover it myself. Perhaps they think it would *injure their business*, if every one was sufficiently informed to keep himself *well*, so they are careful not to tell too much. The presumption is, however, that they do not know themselves. A lady, a member of my father's family, has the Throat Ail. The physician said so, and treated her for it—applied nitrate of silver a few times, and left her cured. Other things were given for the regulation of the system. In a fortnight after she was *cured*, it came on again. Now, *I* believe she has *dyspepsia more* than I have, and *that* is the cause of the throat affection; but the physician never said anything about it; and when asked what was the cause, could not tell."

This letter gives rise to several practical suggestions. *First*, The absurdity of attempting to cure Dyspeptic Throat-Ail, by ramming a swab of nitrate of silver down the throat. *Second*, The commonness of the occurrence of Throat-Ail, or Chronic Laryngitis, from the stomach—the lining of the throat being a continuation of the lining of the stomach. *Third*, The fallacy of the notion, prevalent even among those who ought to have sense enough to know better, that a physician *permits* his patient to keep sick for the sake of the fees, or withholds information purposely from those whom he habitually attends, with a view to their getting sick as often as possible. It is just as much to the highest pecuniary interest of a physician to cure every patient as soon, and as radically as possible, as it is for a mechanic to furnish his customers with a well-made article, or for a merchant who desires to be permanently successful, to sell always a good article of merchandise, to sell it for the quality it is, and no more; that little thing, with not being either afraid or ashamed to do anything which he could do himself, which his business required, even to the sweeping of his eight by ten store, and the carrying his own bundles to his customers, after the "business" of the day was over, were the foundations of the fortune of A. T. STEWART, whose magnificent store on Broadway, opposite the Park, is not surpassed by any dry-goods establishment on the face of the globe.

A *fourth* item is, People are Pigs, for the most part; they are too stupid to want to learn how to keep well, and are too much the slaves of appetite and animal indulgence, to be willing to restrain themselves in eating and drinking, or anything else belonging to the passions. Hence, medical men have found it not worth while to do anything more than to cure their patients. We once advised a gentleman of education and influence, as a means of keeping well of a throat affection, dependent in a large degree on the extravagant use of tobacco, that he must at once and forever break off from the use of it. He wrote in reply: "I can't; I wish the plaguy weed had never been invented." Such people

**Ought to be sick,**
**And to be kicked.**

